# Adaptation of a Protocol for Disclosure of Amyloid PET Results for Adults with Down Syndrome and Their Caregivers

**DOI:** 10.1002/alz70858_100947

**Published:** 2025-12-25

**Authors:** Hayat M Sherif, Lindsay R Clark, Nathaniel A. Chin, Sigan L Hartley, Bradley T Christian, Fred B. Ketchum

**Affiliations:** ^1^ Center for Health Disparities Research, University of Wisconsin‐Madison School of Medicine and Public Health, Madison, WI, USA; ^2^ Wisconsin Alzheimer's Disease Research Center, University of Wisconsin‐Madison School of Medicine and Public Health, Madison, WI, USA; ^3^ Division of Geriatrics and Gerontology, Department of Medicine, University of Wisconsin‐Madison, School of Medicine & Public Health, Madison, WI, USA; ^4^ Wisconsin Alzheimer's Disease Research Center, University of Wisconsin School of Medicine and Public Health, Madison, WI, USA; ^5^ Waisman Center, University of Wisconsin‐Madison, Madison, WI, USA; ^6^ School of Human Ecology, University of Wisconsin–Madison, Madison, WI, USA; ^7^ Department of Medical Physics, University of Wisconsin‐Madison School of Medicine and Public Health, Madison, WI, USA; ^8^ Department of Neurology, University of Wisconsin‐Madison School of Medicine and Public Health, Madison, WI, USA; ^9^ Center for Health Disparities Research, University of Wisconsin School of Medicine and Public Health, Madison, WI, USA

## Abstract

**Background:**

Adults with Down Syndrome (DS) have a greater than 90% lifetime risk of developing dementia due to Alzheimer's Disease (AD). There is an urgent need for clinical trials in this population, which will require biomarker testing and disclosure of results. Disclosure protocols have been widely used for neurotypical individuals. However, effective approaches to disclosure may differ between neurotypical adults at risk for late‐onset AD, and adults with DS who are typically part of a patient‐caregiver dyad with a unique psychosocial context. We developed a protocol to disclose amyloid PET biomarker results for adults with DS and their caregivers participating in the Alzheimer Biomarker Consortium Down Syndrome (ABC‐DS) study.

**Method:**

We adapted a disclosure toolkit that has been extensively tested in longitudinal studies for amyloid PET biomarker disclosure to neurotypical adults. We first identified the unique needs of dyads around disclosure through a literature review and consultations with experts in DS, intellectual and developmental disabilities (IDDs), and AD clinical care. We created a protocol encompassing screening, education, results disclosure, and post‐disclosure check‐in.

**Result:**

Adults with DS are often supported by family caregiver(s), who face challenges including lack of awareness of the increased risk of developing AD, difficulty in obtaining a timely diagnosis, limited resources for psychosocial support, and lack of access to clinical trials. We adapted materials to address the informational needs of dyads and adapted the disclosure protocol to focus on and assess the following: level of understanding of the biomarker results, concerns, and capacity to act on disclosure results and seek resources. Specific training was also provided to disclosing clinicians on the diagnosis of AD in adults with DS.

**Conclusion:**

To our knowledge this is the first adaptation of an amyloid PET disclosure protocol for adults with DS and their caregivers. This adaptation emphasizes empowering the dyad, and is a first step towards achieving equity in AD diagnosis and treatment for a population of individuals with Down Syndrome. In the next phase of research, we will further refine the protocol through pilot testing with adults with DS who have undergone amyloid PET scans as part of ABC‐DS.

